# Effects of estrogens and antiestrogens on gonadal sex differentiation and embryonic development in the domestic fowl (*Gallus gallus domesticus*)

**DOI:** 10.7717/peerj.5094

**Published:** 2018-07-03

**Authors:** Luzie Jessl, Rebecca Lenz, Fabian G. Massing, Jessica Scheider, Jörg Oehlmann

**Affiliations:** 1 Department Aquatic Ecotoxicology, Johann Wolfgang Goethe Universität Frankfurt am Main, Frankfurt am Main, Hesse, Germany; 2 R-Biopharm AG, Darmstadt, Hesse, Germany; 3 Dr. Drexler + Dr. Fecher GmbH, Groß-Umstadt, Hesse, Germany; 4 ERM GmbH, Neu-Isenburg, Hesse, Germany

**Keywords:** Gonad, Chicken embryo, Estrogen, Fulvestrant, Sex differentiation, Antiestrogen, 17α-ethinylestradiol, Tamoxifen, Endocrine disruption

## Abstract

Since it is known that environmental contaminants have the potential to cause endocrine disorders in humans and animals, there is an urgent need for in vivo tests to assess possible effects of these endocrine disrupting chemicals (EDCs). Although there is no standardized guideline, the avian embryo has proven to be particularly promising as it responds sensitively to a number of EDCs preferentially impacting the reproductive axis. In the present study we examined the effects of in ovo exposure to fulvestrant and tamoxifen as antiestrogenic model compounds and co-exposure to both substances and the potent estrogen 17α-ethinylestradiol (EE_2_) regarding sex differentiation and embryonic development of the domestic fowl (*Gallus gallus domesticus*). The substances were injected into the yolk of fertilized eggs on embryonic day 1. On embryonic day 19 sex genotype and phenotype were determined, followed by gross morphological and histological examination of the gonads. Sole EE_2_-treatment (20 ng/g egg) particularly affected male gonads and resulted in an increased formation of female-like gonadal cortex tissue and a reduction of seminiferous tubules. In ovo exposure to tamoxifen (0.1/1/10 µg/g egg) strongly impaired the differentiation of female gonads, led to a significant size reduction of the left ovary and induced malformations of the ovarian cortex, while fulvestrant (0.1/1/10 µg/g egg) did not affect sexual differentiation. However, both antiestrogens were able to antagonize the feminizing effects of EE_2_in genetic males when administered simultaneously. Since both estrogens and antiestrogens induce concentration-dependent morphological alterations of the sex organs, the chick embryo can be regarded as a promising model for the identification of chemicals with estrogenic and antiestrogenic activity.

## Introduction

In recent decades, reproductive disorders in animals and humans and the potential role of chemical substances that are suspected to cause these effects through their endocrine potential became of great interest for science and society. These so-called endocrine disrupting chemicals (EDCs) may alter sex-differentiation and reproduction by very different modes of action. If a chemical substance has the same effects as endogenous sex hormones at the estrogen or androgen receptor, this substance acts as an agonist and its effects are referred to as estrogenic or androgenic. On the contrary it is referred to as antiestrogenic or antiandrogenic when it inhibits the action of endogenous sex hormones as an antagonist at the corresponding steroid receptor. In view of the large number of constantly used chemicals, it is expected that potential EDCs end up in the environment and may affect humans and animals. These chemicals can originate from agriculture or industry, or may be used as pharmaceuticals. In the study of steroidal and non-steroidal substances, e.g., bisphenol A (BPA), 17*α*-ethinylestradiol (EE_2_), tributyltin (TBT) and many more, hormonal effects on different groups of organisms have already been identified (Peakall & Lincer, 1996; Berg et al., 1998; Berg et al., 1999; Watts, Pascoe & Carroll, 2001; Grote et al., 2004; Berg & Pettersson, 2006; Oehlmann et al., 2006; Pettersson et al., 2006; Ahn et al., 2007; Choi et al., 2007; Bodiguel et al., 2009; Scheider et al., 2018; Jessl, Scheider & Oehlmann, 2018). These studies underline the assumption that numerous chemicals have an endocrine potential and may pose a potential threat to the ecosystem and to animal and human health.

In order to assess possible effects and to weigh risks, the testing of chemicals for their endocrine potential is of great importance. So far only a small fraction of the circulating and constantly used chemicals have been tested for a potential effect on the hormonal system. According to REACH (Registration, Evaluation, Authorization and Restriction of Chemicals) further chemicals are to be tested for their harmful potential in Europe. To implement this, a variety of animal experiments have to be executed. For the testing of androgenic and estrogenic EDCs two rodent-based tests, the Hershberger assay (OECD, 2009) and the uterotrophic assay (OECD, 2007), have been internationally standardized. Since mainly juvenile or adult animals with full pain perception are used, the search for a suitable animal replacement system is of great importance. In addition, the developing embryo, which is regarded as the most sensitive stage of life and thus a special subject of protection, is insufficiently considered for the testing of chemicals. Since its development is particularly vulnerable to environmental influences including chemicals, the testing of embryos can unfold possible effects of these substances that may not be detected in adult individuals (Duis et al., 2014).

Beside developmental stages of other animal taxa, avian embryos have been used for a long time to study sexual development and the potential impact of environmental pollutants, including EDCs (Fry & Toone, 1981; Berg et al., 1998; Eising et al., 2001; Berge et al., 2004; Biau et al., 2007). One advantage of working with fertile eggs is that the application of substances, often injected directly into the egg, allows the use of specific and standardized dosages (Berg et al., 1999). As the hen affects the development of its offspring by transferred genetic materials and hormones (Carere & Balthazart, 2007), substances incorporated by the mother may consequently also influence the development of the offspring even originally or as metabolites in the allantoic fluid (Kamata et al., 2006). However, in contrast to developing mammals or aquatic species, the chicken egg is a largely closed system lacking significant exchange with its external environment except for the interchange of gases. However, it should be noted that beyond gas exchange there is still a potential of interaction with the external environment since the embryo is sensitive to changes in temperature and humidity. In addition, the passage of metals (Ackerman et al., 2016) and highly lipophilic organic compounds (Bargar, Scott & Cobb, 2001; Zheng et al., 2014) from mother to offspring has been demonstrated. Thus, one injection of a test compound results in chronic chemical exposure, because no exchange or loss of the substance is possible except for metabolization, protein bonding or further modifications of the substance by the internal embryo environment. A single injection may therefore be sufficient to influence the developing embryo (Davies et al., 1997; Gooding et al., 2003; McAllister & Kime, 2003; Zhang et al., 2007; Scheider et al., 2018; Jessl, Scheider & Oehlmann, 2018).

It is already known that the exposure to xenobiotics during avian embryonic development may cause irreversible malformations of the sex organs and a disruption of gender-specific behavior in adult animals (Adkins-Regan, 1990; Ottinger & Abdelnabi, 1997). The embryo of the domestic fowl (*Gallus gallus domesticus*) is particularly suitable for our experiments as its developmental stages are fully described (Keibel & Abraham, 1900; Hamburger & Hamilton, 1992; Starck & Ricklefs, 1997). However, there is still no standardized procedure for experiments with chicken embryos available.

The present study is part of a project aiming to expedite a protocol to assess the potential effect of EDCs on early sexual differentiation in the chicken embryo. As part of this effort we analyzed the effects of different estrogenic and antiestrogenic compounds on embryonic development with special focus on potential gross morphological and histological changes of the gonads. EE_2_, a synthetic hormone primarily used for contraception was selected for the study of estrogenic substances and was used as a positive substance. It has already been widely used in the study of EDCs and has shown to affect sexual differentiation in bird embryos (Berg et al., 1998; Berg et al., 1999; Berg et al., 2001; Berg et al., 2004; Akazome & Mori, 1999; Watts, Pascoe & Carroll, 2001; Watts, Pascoe & Carroll, 2003; Berg & Pettersson, 2006; Pettersson et al., 2006; Biau et al., 2007; Brunstrom et al., 2009; Scheider et al., 2018; Jessl, Scheider & Oehlmann, 2018). For the testing of antiestrogenic substances tamoxifen and fulvestrant, two well-known drugs with desired hormonal action were selected. Both compounds are used for the first-line endocrine therapy of estrogen receptor-positive metastatic breast cancer (Henderson, 1991; Buzdar, 2001; Buzdar & Robertson, 2006). Furthermore, both have been used for the testing of potential effects on different non-target organisms including the bird embryo (Scheib & Baulieu, 1981; Cevasco et al., 2008; Sun, Zha & Wang, 2009; Hoffmann & Kloas, 2012; Yu et al., 2014).

## Materials and Methods

### Dosing

All experiments were carried out with respect for the principles of laboratory animal care, in accordance with the European Communities Council Directive of 24 November 1986 (86/609/EEC) and the German Animal Welfare Act. Fertilized eggs of white Leghorn (*G. domesticus*) were obtained from a local breeder (LSL Rhein-Main Geflügelvermehrungsbetrieb, Dieburg, Germany). The total number of eggs per experiment and treatment-group are shown in Table 1 for fulvestrant and Table 2 for tamoxifen, including the treatment-groups with parallel sole and co-exposure to 17*α*-ethinylestradiol (EE_2_). The testing of tamoxifen was conducted in a series of four experiments while fulvestrant was tested in a single experiment. Tamoxifen was tested in four experiments to ensure a higher degree of replication for an up to then not tested class of EDCs with unknown effects. The eggs were incubated at 37.5 ± 0.5 °C and 60 ± 10% relative humidity and turned over eight times a day in a fully automated incubator (J. Hemel Brutgeräte, Verl, Germany).

**Table 1:** Mortality (%) and malformations (%) in chicken embryos after in ovo exposure to fulvestrant (Ful, 0.1, 1, 10 µg/g egg) and EE_2_ (20 ng/g egg) or co-exposure to all concentrations of fulvestrant and EE_2_.

Test substance	Fulvestrant			
∑ experiments	1			
∑ eggs	200			
Group	∑ eggs	∑ fertilized eggs	Mortality (%)^a^	Malformations (%)^a^
NC	24	22	18.2 (4)	0.00 (0)
SC	22	20	15.0 (3)	0.00 (0)
Ful 0.1	22	19	26.3 (5)	10.5 (2)
Ful 1	22	22	22.7 (5)	4.55 (1)
Ful 10	22	19	31.6 (6)	5.26 (1)
EE_2_	22	22	45.5 (10)	4.55 (1)
Ful 0.1 +EE_2_	22	20	35.0 (7)	10.0 (2)
Ful 1 +EE_2_	22	22	18.2 (4)	4.55 (1)
Ful 10 +EE_2_	22	18	33.3 (6)	11.1 (2)

**Notes.**

a The number in parentheses represents the number of affected embryos.

TITLE NC untreated control SC solvent control

**Table 2:** Mortality (%) and malformations (%) in chicken embryos after in ovo exposure to tamoxifen (Tam, 0, 1, 1, 10 µg/g egg) and EE_2_ (20 ng/g egg) or co-exposure to all concentrations of tamoxifen (plus 0.001, 0.01 µg/g egg) and EE_2_.

Test substance	Tamoxifen			
∑ experiments	4			
∑ eggs	603			
Group	∑ eggs	∑ fertilized eggs	Mortality (%)^a^	Malformations (%)^a^
NC	93	88	7.95 (7)	0.00 (0)
SC	83	75	28.0 (21)	5.33 (4)
Tam 0.1	60	53	43.4 (23)	5.66 (3)
Tam 1	60	51	39.2 (20)	1.96 (1)
Tam 10	59	52	55.8 (29)	1.92 (1)
EE_2_	47	45	24.4 (11)	6.67 (3)
Tam 0.001 +EE_2_	30	28	7.14 (2)	7.14 (2)
Tam 0.01 +EE_2_	31	30	20.0 (6)	3.33 (1)
Tam 0.1 +EE_2_	46	44	34.1 (15)	4.54 (2)
Tam 1 +EE_2_	47	45	40.0 (18)	4.44 (2)
Tam 10 +EE_2_	47	43	30.2 (13)	6.98 (3)

**Notes.**

a The number in parentheses represents the number of affected embryos.

TITLE NC untreated control SC solvent control

Fulvestrant (CAS: 129453-61-8; purity: ≥98%), tamoxifen (CAS: 10540-29-1; purity: ≥99%) and EE_2_ (CAS: 57-63-6; purity: ≥98%) were purchased from Sigma Aldrich Chemie GmbH (München, Germany). Fulvestrant (applied doses: 0.1, 1, 10 µg/g egg), tamoxifen (applied doses: 0.001, 0.01, 0.1, 1, 10 µg/g egg) and EE_2_ (applied dose: 20 ng/g egg) were dissolved alone or in combination in 60 µL of the solvent dimethyl sulfoxide (DMSO; CAS: 67-68-5; purity: 99.5%; Applichem, Darmstadt, Germany). Eggs were yolk-injected on day 1 of incubation and further processed until dissection as described elsewhere (Jessl, Scheider & Oehlmann, 2018).

### Dissection, tissue preparation and evaluation

On day 19 of incubation embryos were dissected. Deformations of body and internal organs were recorded with special focus on ovaries and testes. Gonads were photographed (Diskus, Carl H. Hilgers, Königswinter, Germany) for further analysis of the gonad surface area, in which the entire visible surface of each single gonad was determined with an image editing program (Fiji is just ImageJ, Open Source). After dissection gonads were fixed in Bouin’s solution, which was removed by repeated rinsing with 80% ethanol after 24 h. Ethanol was removed by saccharose solution (10, 20 and 30% in phosphate buffered saline). Gonads were embedded in Tissue-Tek^®^ (Sakura Finetek Europe B.V., Alphen aan den Rijn, Netherlands) and sectioned (6 µm) by a freeze microtome (Microm HM 500 O, Thermo Fisher Scientific Germany, Bonn, Germany) at −23 °C. Tissue sections were stained with hematoxylin and eosin.

### Measurements and statistics

Histological examination was performed using a light microscope (Olympus BX50, Olympus, Tokyo, Japan) and a camera (JVC Digital Camera, KY-F75U, Yokohama, Japan). Gonadal cortex thickness of both sexes and male percentage of seminiferous tubules in left testes were measured (Fiji is just ImageJ, Open Source). Ten sections for each embryo were evaluated which were exclusively taken from the gonad’s middle sectional plane as described previously (Jessl, Scheider & Oehlmann, 2018).

For the test series with the combination of tamoxifen and EE_2_, the results of four test runs and for the combination of fulvestrant and EE_2_, the results of a single test run were merged and analyzed. For the endpoints, gonadal cortex thickness and percentage of seminiferous tubules as well as gonad surface area data were normalized to the solvent control. Data were analyzed using Fisher’s exact test, one-way ANOVA with Dunnett’s multiple comparison test or Kruskal-Wallis test with Dunn’s multiple comparison test with GraphPad Prism 5.01 (GraphPad Software Inc., San Diego, USA).

### Determination of sexual genotype

DNA isolation was performed using a tissue sample from the heart taken during dissection. All embryos were typed for their sexual ZZ or ZW genotype, using the PCR-based method of Fridolfsson & Ellegren (1999) with the primers 2550F (5′-GTT ACT GAT TCG TCT ACG AGA-3′) and 2718R (5′-ATT GAA ATG ATC CAG TGC TTG-3′) and a modified protocol. Both primers mark two CHD1 introns, located on the Z (CHD1Z, 600 bp) and W chromosome (CHD1W, 450 bp). Thermal cycling was composed of DNA polymerase activation at 95 °C for 15 min followed by 40 cycles of denaturation at 95 °C for 30 s, annealing at 52 °C for 30 s, elongation at 72 °C for 40 s and a final extension step at 72 °C for 5 min followed by a melt curve (60–95 °C with a heating rate of 0.2 °C/s). Following qPCR-mediated amplification with EvaGreen^®^ dye, all amplicons showed two different melting peaks based upon the different melting properties of double-stranded DNA, which resulted in characteristic bands for each sex (Chen et al., 2012). Both sexes had a single 600-bp CHD1-Z specific fragment with a melting temperature of ∼84 °C. Females had an additional 450-bp CHD1-W female-specific fragment with a melting temperature of ∼82 °C.

## Results

### Effects of in ovo exposure to fulvestrant and EE_2_

#### Embryonic mortality and malformations

**Figure 1:**
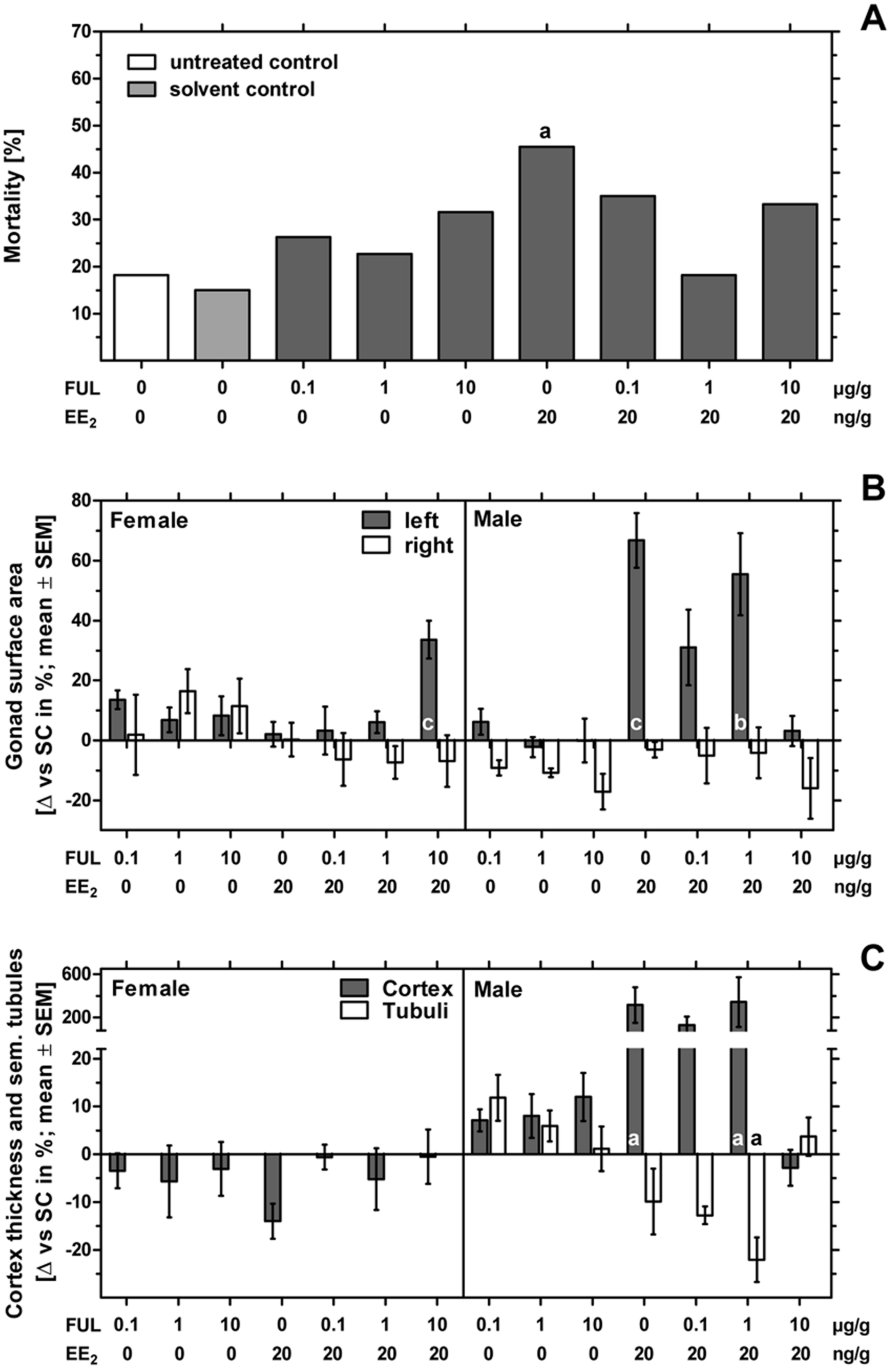
Effects of in ovo exposure to fulvestrant and 17*α*-ethinylestradiol on mortality, left and right gonad surface area, cortex thickness and percentage of seminiferous tubules of left gonad of embryos of the domestic fowl (*Gallus g. domesticus*). Effects of in ovo exposure to fulvestrant (FUL, 0.1, 1, 10 µg/g egg) and 17*α*-ethinylestradiol (EE_2_; 20 ng/g egg) on mortality (A), left and right gonad surface area (B) and cortex thickness and percentage of seminiferous tubules of left gonad (C) of embryos of the domestic fowl (*Gallus g. domesticus*) on embryonic day 19. Statistical analysis by Fisher’s exact test (A) and one-way ANOVA with Dunnett’s multiple comparison test (B, C). Grey background distinguishes the co-exposure to fulvestrant and EE_2_. Lowercase indicate significant differences compared to the solvent control. Level of significance: a: *p* < 0.05; b: *p* < 0.01; c: *p* < 0.001.

The number of fertilized eggs, embryonic mortality and malformations per treatment-group are presented in Table 1. The fertility rate of the individual groups was at least 82% with a mean total fertility rate of 92% for the whole experiment. The solvent control group showed the lowest mortality of 15%, followed by the untreated control group with 18% mortality. Sole treatment to fulvestrant as well as co-exposure to all concentrations of fulvestrant plus EE_2_ showed mortality rates up to 35%. Sole treatment to EE_2_ caused 46% mortality being significantly different from the control (*p* < 0.05) (Fig. 1A).

While no malformations were detected in the control groups, all substance treated groups showed one to two malformed embryos. The EE_2_-treated group 1 of 22 embryos (4.55%) showed a single malformation which was found to be celosomia. Examining all groups receiving different concentrations of fulvestrant, four of 60 embryos (6.67%) showed malformations which were mainly found to be celosomia or malformations of the eyes or the beak. Examining all groups co-exposed to fulvestrant and EE_2_, five of 60 embryos (8.33%) showed malformations which were mainly found to affect the beak, the eyes, the limbs, the brain or celosomia. Compared to the control, none of the tested groups showed a substance-induced increase in the rate of malformation.

#### Morphological observation of the gonads—gonad surface area

In females sole exposure to EE_2_ or all concentrations of fulvestrant as well as co-exposure to EE_2_ and lower concentrations of fulvestrant (0.1 and 1 µg/g egg) had no statistically significant effect on left and right gonad surface area. Co-exposure to EE_2_ and 10 µg fulvestrant/g egg, however, resulted in a statistically significant increase of the left gonad surface area (*p* < 0.001) (Fig. 1B and Table 3).

**Table 3:** Gonad surface area, gonadal cortex thickness and percentage of seminiferous tubules of chicken embryos after in ovo exposure to fulvestrant (Ful, 0.1, 1, 10 µg/g egg) and EE_2_ (20 ng/g egg) or co-exposure to all concentrations of fulvestrant and EE_2_.

Sex	Group	Gonad surface area		Cortex thickness (µm)	Seminiferous tubules (%)
		left (mm^2^)	right (mm^2^)		
Male	NC	4.22 ± 0.95	3.92 ± 0.57	10.1 ± 0.40	28.3 ± 4.49
	SC	3.99 ± 0.46	3.91 ± 0.30	10.7 ± 0.73	30.2 ± 1.83
	Ful 0.1	4.24 ± 0.42	3.55 ± 0.24	11.5 ± 0.61	33.8 ± 3.56
	Ful 1	3.90 ± 0.36	3.49 ± 0.13	11.6 ± 1.30	32.0 ± 2.60
	Ful 10	3.99 ± 0.82	3.24 ± 0.66	12.0 ± 1.54	30.6 ± 3.98
	EE_2_	6.66 ± 0.82^c^	3.79 ± 0.23	44.6 ± 39.3^a^	27.3 ± 4.65
	Ful 0.1 +EE_2_	5.23 ± 1.23	3.71 ± 0.89	25.0 ± 18.3	26.4 ± 1.23
	Ful 1 +EE_2_	6.21 ± 1.09^b^	3.75 ± 0.67	47.6 ± 49.4^a^	23.6 ± 2.82^a^
	Ful 10 +EE_2_	4.12 ± 0.53	3.29 ± 1.05	10.4 ± 0.91	31.4 ± 2.70
Female	NC	10.3 ± 0.69^c^	2.38 ± 0.34^b^	143 ± 33.9	–
	SC	8.23 ± 0.95	1.80 ± 0.34	154 ± 30.0	–
	Ful 0.1	9.35 ± 0.72	1.84 ± 0.68	149 ± 15.0	–
	Ful 1	8.80 ± 1.03	2.10 ± 0.40	145 ± 32.7	–
	Ful 10	8.91 ± 1.20	2.01 ± 0.37	149 ± 19.5	–
	EE_**2**_	8.40 ± 0.91	1.81 ± 0.27	133 ± 15.0	–
	Ful 0.1 +EE_2_	8.50 ± 1.98	1.69 ± 0.45	153 ± 8.89	–
	Ful 1 +EE_2_	8.74 ± 1.12	1.67 ± 0.37	146 ± 37.2	–
	Ful 10 +EE_2_	11.0 ± 1.37^c^	1.68 ± 0.41	153 ± 21.5	–

**Notes.**

Statistical analysis by one-way ANOVA with Dunnett’s multiple comparison test. Lowercase indicate significant differences compared to the solvent control (SC). NC, untreated control. Level of significance: ^a^*p* < 0.05; ^*b*^*p* < 0.01; ^*c*^0.001.

**Figure 2:**
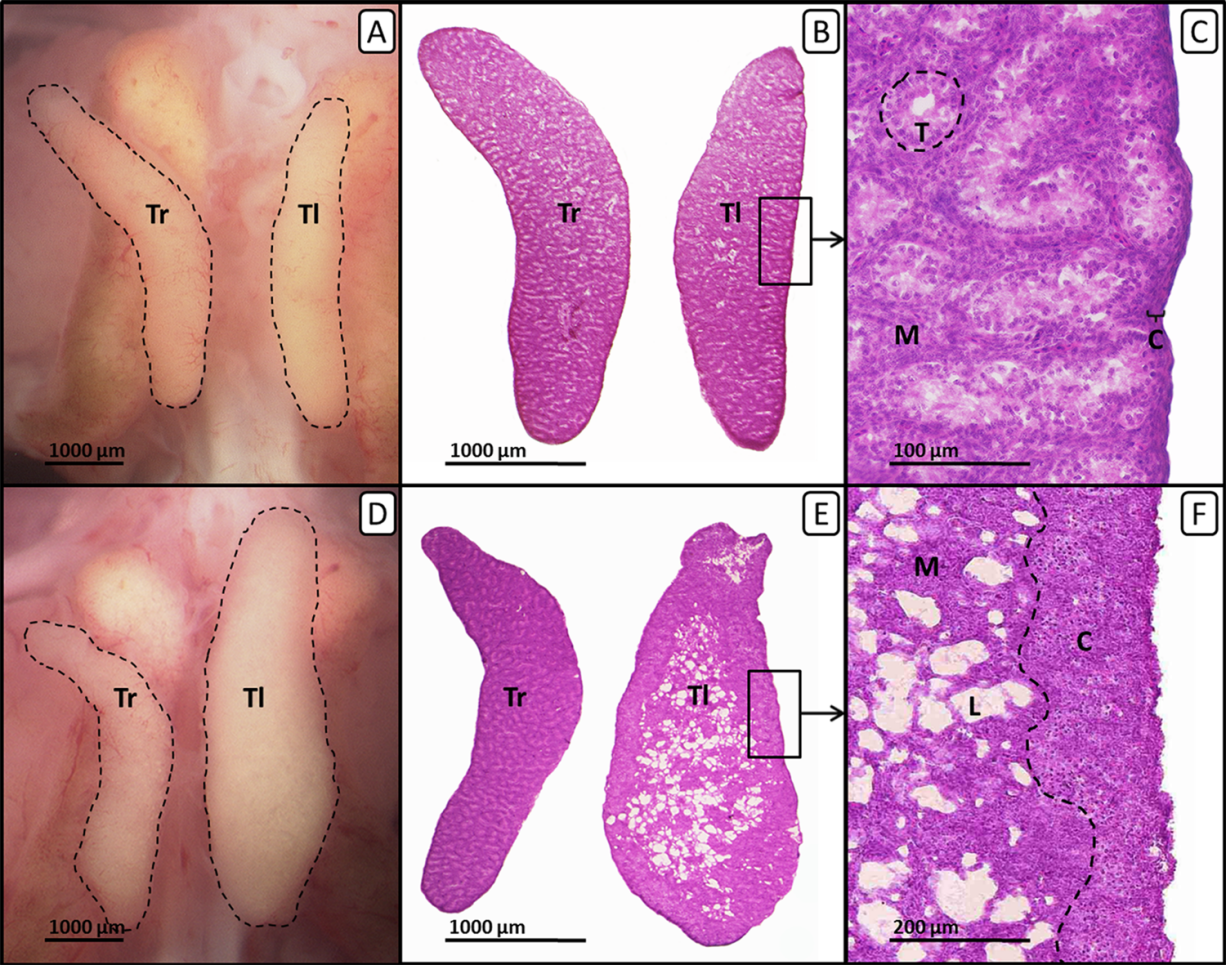
Right and left testis of genetic males of untreated control group and EE_2_-treated group on day 19 of embryonic development. Right and left testis of genetic males of untreated control group (A, B, C) and EE_2_-treated group (20 ng/g egg; D, E, F). (A, D) Unfixed right (“Tr”) and left (“Tl”) testis (outlined in black) on day 19 of embryonic development. Untreated control males (A) show testes of nearly identical size. EE_2_-treated males (D) show an unaffected right testis and a significantly enlarged left testis. (B, E) Histological thin sections (6 µm) of right (“Tr”) and left (“Tl”) testis. Untreated control males (B) show testes of nearly identical size and structure. EE_2_-treated males (E) show an unaffected right testis and a significantly enlarged left testis with female-like shape and structure. (C, F) Histological thin sections (6 µm) of left testis in close-up. Untreated control males (C) show a thin gonadal cortex layer (“C”) of three to four cells and interstitial space and seminiferous tubules (“T”) in the medulla (“M”). Left testes of EE_2_-treated males (F) show female-typical structures as a well-differentiated gonadal cortex region (“C”) with oocyte-like cells and a loosely arranged medulla (“M”) crossed by lacunar channels (“L”).

In males fulvestrant did not affect left and right gonad surface area at all applied sole concentrations. EE_2_ administered alone or in combination with lower concentrations of fulvestrant (1 µg/g egg) resulted in a significant increase in the left gonad surface area (*p* < 0.001 and *p* < 0.01, respectively). The affected left testes showed a female-like shape and a well visible female-typical thickened cortex region when viewed under a stereomicroscope (Fig. 2). However, a concentration of 10 µg fulvestrant/g egg completely antagonized the EE_2_-induced feminization of genetic males.

#### Histological observation of the gonads—left testis and ovary

In females sole exposure to all concentrations of fulvestrant or co-exposure to fulvestrant and EE_2_ had no statistically significant effect on ovarian cortex thickness (Fig. 1C).

Administration of EE_2_ alone or in combination with 1 µg fulvestrant/g egg caused a significant increase in male gonadal cortex thickness by up to 344% (*p* < 0.05, respectively) while the percentage of seminiferous tubules decreased with increasing concentration of fulvestrant (co-exposure to EE_2_ and 1 µg fulvestrant/g egg: *p* < 0.05). The affected testicular tissue appeared significantly changed with female-typical structures such as lacunae and a differentiated female-like gonadal cortex region with oocyte-like cells. On the contrary, the number and degree of differentiation of seminiferous tubules were visibly lower. A concentration of 10 µg fulvestrant/g egg completely antagonized the feminization of genetic males caused by EE_2_ (Table 3).

### Effects of in ovo exposure to tamoxifen and EE_2_

#### Embryonic mortality and malformations

**Figure 3:**
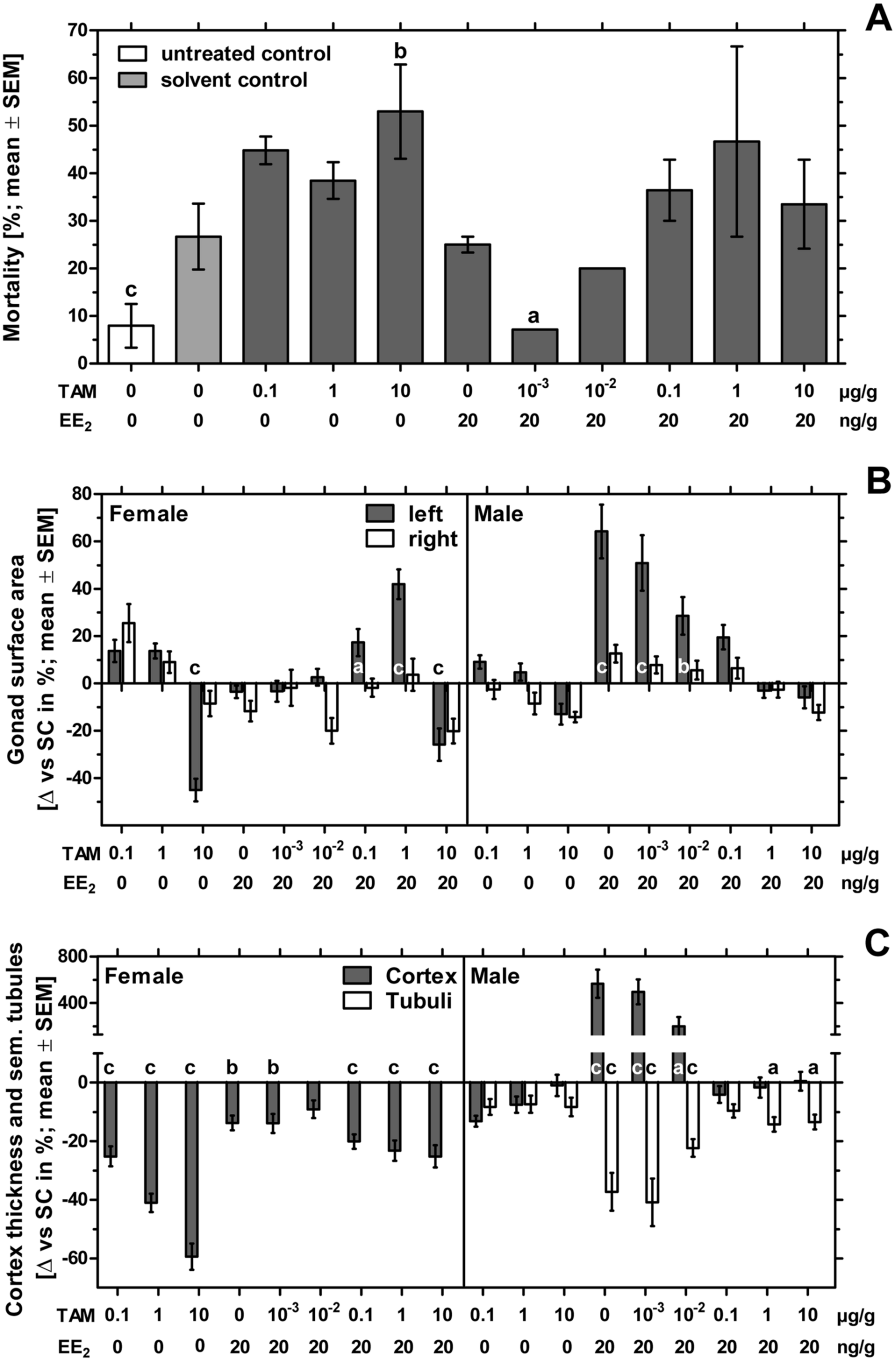
Effects of in ovo exposure to tamoxifen and 17*α*-ethinylestradiol on mortality, left and right gonad surface area, cortex thickness and percentage of seminiferous tubules of left gonad of embryos of the domestic fowl (*Gallus g. domesticus*). Effects of in ovo exposure to tamoxifen (TAM, 0.001, 0.01, 0.1, 1, 10 µg/g egg) and 17*α*-ethinylestradiol (EE_2_, 20 ng/g egg) on mortality (A), left and right gonad surface area (B) and cortex thickness and percentage of seminiferous tubules of left gonad (C) of embryos of the domestic fowl (*Gallus g. domesticus*) on embryonic day 19. Statistical analysis by Fisher’s exact test (A), one-way ANOVA with Dunnett’s multiple comparisons test or Kruskal-Wallis test with Dunn’s multiple comparison test (B, C). Grey background distinguishes the co-exposure to tamoxifen and EE_2_. Lowercase indicates significant differences compared to the solvent control. Level of significance: a, *p* < 0.05; b, *p* < 0.01; c, *p* < 0.001.

The number of fertilized eggs, embryonic mortality and malformations per treatment-group are presented in Table 2. The fertility rate of the individual groups was at least 88% with a total fertility rate of 92% for the whole experiment. The unmanipulated negative control showed the lowest mortality of nearly 8% and differed significantly from the solvent-treated control with 28% mortality (*p* < 0.001). Sole exposure to tamoxifen showed mortality rates up to 56%. Only the group treated with 10 µg tamoxifen/g egg showed a significant difference to the solvent-treated control (*p* < 0.01). Sole exposure to 20 ng EE_2_/g egg caused 24% mortality. Groups co-exposed to tamoxifen and EE_2_ showed mortality rates up to 40% with a significant deviation to the solvent-treated control for the group co-exposed to EE_2_ and 0.001 µg tamoxifen/g egg (*p* < 0.05) (Fig. 3A).

While no malformations were detected in the untreated negative control group, four of 75 embryos (5.33%) of the solvent-treated control showed malformations which affected the eyes, the beak, the extremities or edema or celosomia. In the EE_2_-treated group three of 45 embryos (6.67%) showed malformations of the eyes, the beak or celosomia. Examining all groups receiving different concentrations of tamoxifen, five of 156 embryos (3.21%) showed malformations which were found to be celosomia, malformations of the vertebral column, the beak or the brain. There was also one egg containing two embryos conjoined at the head, showing various malformations. Examining all groups co-exposed to EE_2_ and different concentrations of tamoxifen, 10 of 190 embryos (5.26%) showed malformations which were found to be celosomia, malformations of the beak or the extremities or a missing mesonephros. Compared to the untreated negative control group, the solvent-treated group and the substance-treated groups receiving EE_2_ or a combination of EE_2_ and 10 µg tamoxifen/g egg showed a statistically significant effect (*p* < 0.05). On the contrary, there were no significant differences between the substance-treated groups and the solvent control.

#### Morphological observation of the gonads—gonad surface area

In females exposure to 10 µg tamoxifen/g egg resulted in a statistically significant decrease of the left gonad surface area (*p* < 0.001) (Fig. 3B). This effect could not be antagonized by the simultaneous administration of EE_2_. The combination of EE_2_ and 0.1 or 1 µg tamoxifen/g egg resulted in a statistically significant increase in the left gonad surface area (*p* < 0.05 and *p* < 0.001, respectively). Female right gonad surface areas were not significantly affected by sole exposure to EE_2_ or tamoxifen or the co-exposure to tamoxifen and EE_2_.

In comparison, male left and right gonad surface area were not significantly affected by sole exposure to all concentrations of tamoxifen. The EE_2_-induced statistically significant increase of the male left gonad surface area (*p* < 0.001) could be gradually reduced through simultaneous administration of increasing tamoxifen-concentrations. From a concentration of 0.1 µg tamoxifen/g egg EE_2_-related effects were largely antagonized and left gonad surface areas fluctuated around the control values (Table 4).

**Table 4:** Gonad surface area, gonadal cortex thickness and percentage of seminiferous tubules of chicken embryos after in ovo exposure to tamoxifen (Tam, 0.1, 1, 10 µg/g egg) and EE_2_ (20 ng/g egg) or co-exposure to all concentrations of tamoxifen (plus 0.001, 0.01 µg/g egg) and EE_2_.

Sex	Group	Gonad surface area		Cortex thickness (µm)	Seminiferous tubules (%)
		left (mm^2^)	right (mm^2^)		
Male	NC	3.96 ± 0.65	3.67 ± 0.58^a^	8.67 ± 0.96^b^	28.7 ± 3.11
	SC	3.80 ± 0.61	3.35 ± 0.63	9.35 ± 1.08	29.8 ± 3.29
	Tam 0.1	4.15 ± 0.41	3.26 ± 0.53	8.12 ± 0.61	27.3 ± 2.90
	Tam 1	3.99 ± 0.54	3.06 ± 0.60	8.65 ± 1.00	27.6 ± 3.09
	Tam 10	3.31 ± 0.50	2.87 ± 0.22	9.26 ± 0.84	27.3 ± 2.51
	EE_2_	6.25 ± 1.67^c^	3.77 ± 0.48	62.3 ± 45.6^c^	18.7 ± 8.16^c^
	Tam 0.001 +EE_2_	5.74 ± 1.67^c^	3.61 ± 0.45	55.7 ± 34.6^c^	17.6 ± 8.38^c^
	Tam 0.01 +EE_2_	4.89 ± 1.04^b^	3.54 ± 0.46	28.2 ± 22.1^a^	23.1 ± 2.69^c^
	Tam 0.1 +EE_2_	4.55 ± 0.70	3.56 ± 0.53	8.97 ± 0.96	26.9 ± 2.42
	Tam 1 +EE_2_	3.69 ± 0.42	3.27 ± 0.42	9.19 ± 1.19	25.5 ± 2.79^a^
	Tam 10 +EE_2_	3.58 ± 0.80	2.94 ± 0.49	9.40 ± 1.25	25.8 ± 3.11^a^
Female	NC	10.2 ± 1.48^b^	2.20 ± 0.41	155 ± 11.4	–
	SC	9.25 ± 1.32	1.99 ± 0.46	153 ± 15.7	–
	Tam 0.1	10.5 ± 1.79	2.49 ± 0.64	115 ± 20.7^c^	–
	Tam 1	10.5 ± 1.31	2.16 ± 0.39	90.5 ± 20.3^c^	–
	Tam 10	5.08 ± 1.71^c^	1.82 ± 0.41	70.4 ± 17.1^c^	–
	EE_2_	8.92 ± 0.95	1.75 ± 0.35	132 ± 13.7^b^	–
	Tam 0.001 +EE_2_	8.93 ± 1.42	1.95 ± 0.52	132 ± 16.4^b^	–
	Tam 0.01 +EE_2_	9.49 ± 1.14	1.59 ± 0.36	139 ± 16.1	–
	Tam 0.1 +EE_2_	10.8 ± 2.13^a^	1.95 ± 0.30	123 ± 14.5^c^	–
	Tam 1 +EE_2_	13.1 ± 2.09^c^	2.06 ± 0.48	118 ± 17.8^c^	–
	Tam 10 +EE_2_	6.86 ± 2.00^c^	1.59 ± 0.31	115 ± 18.3^c^	–

**Notes.**

Statistical analysis by one-way ANOVA with Dunnett’s multiple comparison test or Kruskal Wallis test with Dunn’s post test (female right gonad surface area). Lowercase indicate significant differences compared to the solvent control (SC). NC, untreated control. Level of significance: ^a^*p* < 0.05; ^b^*p* < 0.01; ^c^*p* < 0.001.

#### Cortex thickness and percentage of seminiferous tubules

**Figure 4:**
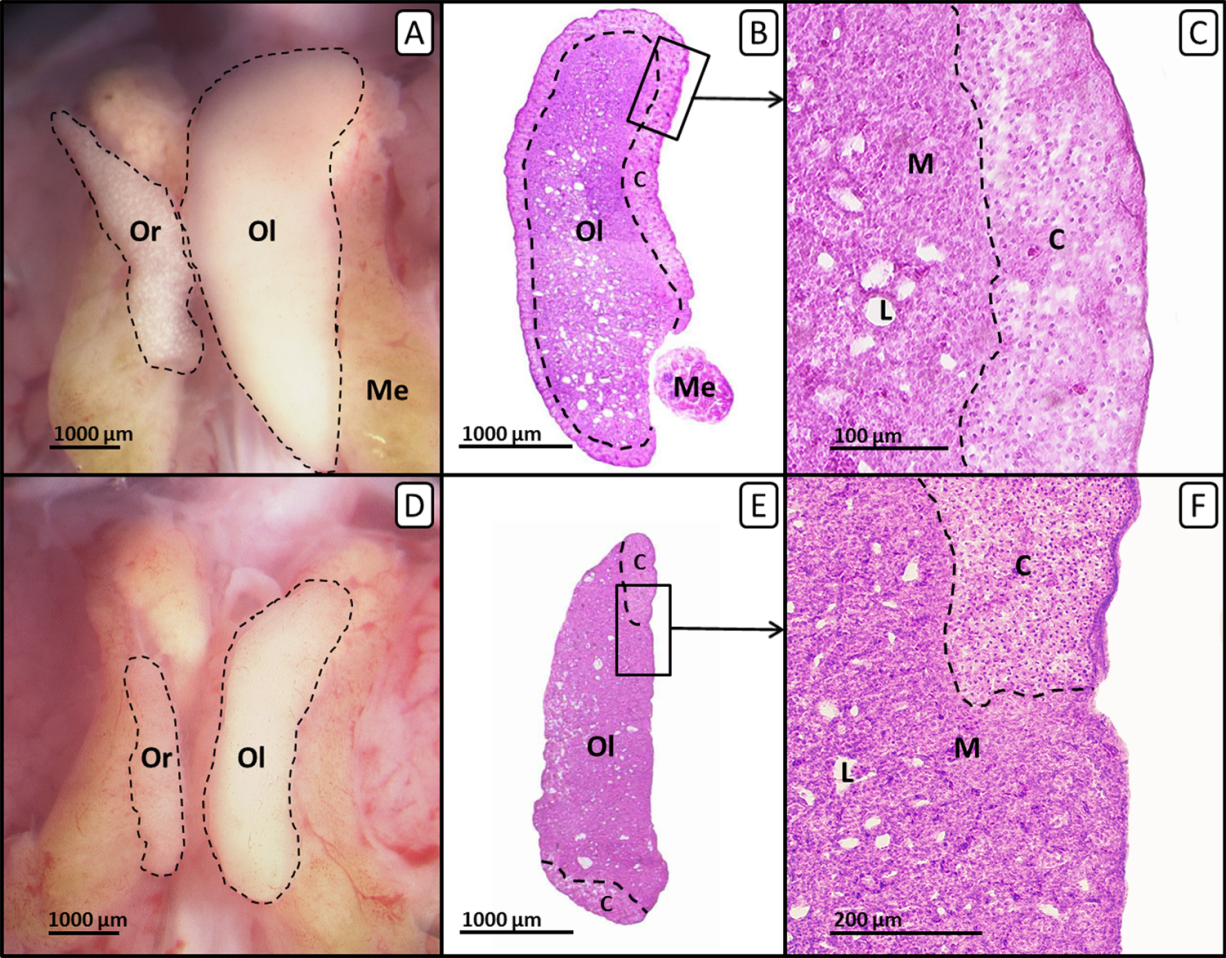
Right and left ovary of genetic females of untreated control group and tamoxifen-treated group on day 19 of embryonic development. Right and left ovary of genetic females of untreated control group (A, B, C) and tamoxifen-treated group (10 µg/g egg; D, E, F). (A, D) Unfixed right (“Or”) and left (“Ol”) ovary (outlined in black) on day 19 of embryonic development. Untreated control females (A) show a regressed right ovary and a well-differentiated left ovary. Tamoxifen-treated females (D) show an unaffected right ovary and a significantly decreased left ovary. (B, E) Histological thin sections (6 µm) of right (“Or”) and left (“Ol”) ovary. Untreated control females (B) show a left ovary of female typical size and structure. The left ovarian cortex (“C”) is well-differentiated and covers almost the whole ovary (except the region close to the mesonephron (“Me”)). Tamoxifen-treated females (E) show a significantly decreased left ovary with an altered distribution of the cortex region (“C”). The left ovary is no longer covered by a continuous cortex but exposes large regions of uncovered medulla, partly resembling a male testis. (C, F) Histological thin sections (6 µm) of the left ovary in close-up. Untreated control females (C) show a well-differentiated continuous ovarian cortex region (“C”) and a loosely arranged medulla (“M”) crossed by lacunar channels (“L”). Tamoxifen-treated females (F) show a discontinuous irregular scattered ovarian cortex region (“C”) with larger regions of uncovered medulla (“M”) crossed by lacunar channels (“L”).

In ovo exposure to all concentrations of tamoxifen as well as co-exposure to tamoxifen and EE_2_ resulted in a statistically significant concentration-dependent decrease of the left ovarian cortex thickness in females (Fig. 3C). Remarkably, several females exposed to the highest concentration of tamoxifen (10 µg/g egg) also showed an altered distribution of the ovarian cortex region (Fig. 4), which could not be detected at lower concentrations. The affected left ovaries were no longer covered by a continuous ovarian cortex but exhibited larger regions of uncovered medulla, partly resembling a testis. In none of any other experiments, even with different endocrine active compounds, was such a phenomenon detected (Scheider et al., 2014; Scheider et al., 2018; Jessl, Scheider & Oehlmann, 2018).

In males the testicular cortex thickness or the percentage of seminiferous tubules were not affected by sole tamoxifen-exposure. Administration of EE_2_ alone or in combination with lower concentrations of tamoxifen (0.001 and 0.01 µg/g egg) resulted in a statistically significant increase in testicular cortex thickness by up to 566%, which was completely suppressed at higher concentrations of tamoxifen (0.1, 1 and 10 µg/g egg). By administration of EE_2_ alone or in combination with lower concentrations of tamoxifen (0.001, 0.01 µg/g egg) the percentages of seminiferous tubules were significantly decreased (*p* < 0.001). However, higher concentrations of tamoxifen (0.1, 1 and 10 µg/g egg) could partly (percentage of seminiferous tubules) or completely (testicular cortex thickness) antagonize the EE_2_-induced feminization of genetic males (Table 4).

## Discussion

### Embryonic mortality and malformations

In the present study fertilization rates were comparable for all experiments with values around 90%. These values are consistent with the results of Romanoff & Romanoff (1972), which described a fertilization rate of almost 89% in an experiment examining 202 breeding hens. The detection of unfertilized eggs is of importance here since the calculation of mortality and the incidence of malformations is based on the number of fertile eggs. While the untreated control showed mortality rates below 20% in all experiments, in 80% (four of five) of the experiments the mortality in the solvent control was below 30%. Based on the data of various publications (Romanoff & Romanoff, 1972; Wyatt & Howarth, 1976; DeWitt, Meyer & Henshel, 2005a; DeWitt, Meyer & Henshel, 2005b) and our own investigations (Jessl, Scheider & Oehlmann, 2018), the natural mortality of unmanipulated chicken embryos is expected to be about 20%. Since it is known that carrier substances in general, or DMSO in the present case, can affect the survival of chicken embryos, for example through their intrinsic toxicity, volume, or density, an increase in mortality compared to the untreated control was expected (Caujolle et al., 1967; Carew & Foss, 1972; Landauer & Salam, 1972; Morgan, 1974; Wyatt & Howarth, 1976). Not only in the present study (110 untreated and 95 solvent-treated individuals in five experiments), but also in further investigations (Jessl, Scheider & Oehlmann, 2018; 256 untreated and 258 solvent-treated individuals in 15 experiments), all of the experiments showed mortality rates below 20% for the untreated control and a predominant proportion of the experiments (87%; 13 of 15 experiments) showed mortality rates below 30% for the solvent control. Due to the large sample size and the consistent results, we assume that the shown values can be reliably used for the comparison to substance-treated groups.

In general, the treatment of chicken embryos with any of the compounds fulvestrant or tamoxifen alone or in combination with EE_2_ led to an increase in mortality as expected. However, significant deviations in mortality from the solvent control were rare and found to be concentration-independent. Groups treated with a defined concentration of fulvestrant or tamoxifen responded similarly to groups that were co-exposed to the same substance plus EE_2_. The absence of a statistically significant difference between solely treated and co-exposed groups of the same substance and concentration suggests that the additional administration of EE_2_ does not necessarily result in an increase in mortality, as one might have expected. Despite the increase in mortality in some groups of the experiments, a sufficient number of vital embryos remained for subsequent histological studies.

Analyzing the frequency of malformations in the five experiments, no malformations were found in the untreated control group with 110 embryos while in the solvent-treated control group four of 95 embryos (4.21%) exhibited malformations. To some extent, this statistically significant increase in the rate of malformation from untreated to solvent-treated control group reflects a weak teratogenic potential of DMSO as noted by Wyatt & Howarth (1976). However, this value is still in the range of the reported spontaneous malformation rate in chicken embryos of 2–7% (Alsop, 1919; Byerly, 1930; Caujolle et al., 1967). In this context, the incidence of malformations in the solvent-treated control can be considered as inconspicuous.

Since there are no statistically significant differences in the frequency of malformations between the individual concentrations of the respective substances fulvestrant, tamoxifen and/or EE_2_ in all experiments, they are combined in groups representing the respective substance (EE_2total,_ fulvestrant_total_, fulvestrant + EE_2total_, tamoxifen_total_ and tamoxifen + EE_2total_). While almost all of the substance groupings show significantly higher malformation rates than the untreated control (EE_2total,_ fulvestrant_total_ and tamoxifen + EE _2total_: *p* < 0.05; fulvestrant + EE _2total_: *p* < 0.01), none of them are significantly different from the solvent-treated control. In addition, there are no statistically significant differences between the respective substance groupings. However, some of the substance-treated groupings show a marginally (EE_2total_, tamoxifen + EE_2total_; *p* > 0.05) or significantly (fulvestrant_total_: *p* < 0.05) increased incidence of celosomia, compared to the solvent-treated control. In addition, exencephalia are exclusively found in substance-treated groups, while the formation of edema is completely absent. Various malformations have already been described, among them, for example, terata of the eyes, the beak, the brain or the formation of celosomia (Byerly, 1930; Caujolle et al., 1967). These terata largely coincide with the malformations found in the solvent- and substance-treated groups. There is no statistical evidence that treatment with fulvestrant, tamoxifen and/or EE_2_ specifically favors particular terata or generally results in increased malformation rates.

### Morphological observations of the gonads—gonad surface area

Since it is known that substances with endocrine potential are able to induce morphological changes in the sex organs of birds (Scheib, 1983; Berg et al., 1998; Berg et al., 1999; Berg et al., 2001; Berg, Halldin & Brunstrom, 2001; Matsushita et al., 2006; Razia et al., 2006), the chicken embryo appears as a suitable model for the study of early sexual development and the potential impact of EDCs impacting the reproductive axis. In birds, the genetic male is homozygous (ZZ), while the genetic female is heterozygous (ZW). During embryonic development the differentiation in one of the sexes depends on the level of circulating steroid hormones, mainly estrogen (Bruggeman, Van As & Decuypere, 2002). Without estrogen or external influences the undifferentiated gonads of genetic males develop into testes. In genetic females, the key enzyme P450 aromatase (P450arom) is synthesized and testosterone is metabolized to estrogen (Kagami & Hanada, 1997; Ayers, Sinclair & Smith, 2013; Scheider et al., 2014). Since P450arom is not synthesized in male gonads (Ayers, Sinclair & Smith, 2013; Scheider et al., 2014), constitutional estrogen concentration in testes is very low (Woods & Erton, 1978; Tanabe et al., 1979; Tanabe, Yano & Nakamura, 1983) and not sufficient to cause a feminization. Though, for a short time during embryonic development the estrogen receptor (ER) is detectable in male gonads, making males basically vulnerable to estrogen (Gasc, 1980; Smith, Andrews & Sinclair, 1997; Nakabayashi et al., 1998). The artificial presence of estrogen or estrogen-active EDCs at this critical time point therefore causes the differentiation of genetic males towards the phenotypically female sex as demonstrated in the present study and various other studies on domestic fowl (*Gallus g. domesticus*) and japanese quail (*Coturnix japonica*) (Sotonyi & Csaba, 1986; Etches & Kagami, 1997; Berg et al., 1998; Berg et al., 1999; Berg et al., 2001; Berg, Halldin & Brunstrom, 2001; Shibuya et al., 2004).

Furthermore, it is shown that the injection of interfering substances into fertilized eggs, such as aromatase inhibitors, can result in a stop of estrogen synthesis leading to a masculinization of ovaries (Elbrecht & Smith, 1992; Burke & Henry, 1999). Not only aromatase inhibitors, but also antiestrogens are capable of affecting female gonad differentiation in birds. Tamoxifen, a selective ER modulator, interacts with various proteins involved in the transcription of estrogen-regulated genes (MacGregor & Jordan, 1998). In the present study sole exposure to high concentrations of tamoxifen resulted in a significant decrease of the left ovarian surface area. This data coincide with the studies of Scheib (1983) on quail embryos, which show that treatment with tamoxifen reduces the size of the left ovary and disturbs the correct formation of the left ovarian cortex. Surprisingly, sole exposure to all concentrations of fulvestrant had no such effect on female ovarian surface area. Due to its considerably higher affinity to ER compared to tamoxifen (Wakeling & Bowler, 1987; Wakeling, Dukes & Bowler, 1991) and the fact that fulvestrant-treatment results in a complete inhibition of the estrogen signaling pathway (Osborne et al., 1995; Wakeling, 1995; Wardley, 2002) we expected fulvestrant as the more potent antiestrogen. The difference between fulvestrant and tamoxifen regarding their effect on embryonic ovarian development of *Gallus domesticus* could be due to their mode of action, since tamoxifen is a partial ER antagonist, depending on the target tissue, and thus has both antiestrogenic and estrogen-like activity while fulvestrant is a pure or competitive ER antagonist without intrinsic activity (Webb et al., 1995; MacGregor & Jordan, 1998; Bentrem et al., 2001; Shou et al., 2004). However, it is unknown whether these differences can impact the activity in ovo.

The present study also demonstrates a concentration-dependent neutralization of the feminizing effect of EE_2_ with increasing concentration of fulvestrant or tamoxifen. In genetic females co-exposure to EE_2_ and higher concentrations of fulvestrant or tamoxifen led to a statistically significant increase in female left gonad surface area. This data coincide with the study of Scheib (1983) which shows that the feminizing effect of diethylstilbestrol on male quail embryos could be largely compensated by simultaneous tamoxifen-treatment. While in the experiments of Scheib (1983) sole treatment with the estrogenic compound diethylstilbestrol reduces the volume of the left ovary but does not affect its differentiation, co-exposure to tamoxifen and the estrogen results in a reduction of the tamoxifen-related effects in females. It can be assumed that the competition between estrogen and antiestrogen at the binding site of the ER is influenced by their differing concentrations but also by their specific binding affinity to the receptor. Blair et al. (2000) reported relative binding affinities to the ER from rat uterine cytosol preparations of 190%, 1.6% and 37.5% for EE_2_, tamoxifen and fulvestrant, respectively. Because both antiestrogens were applied at doses up to factor 500 higher than the estrogen, it is likely that the estrogen is successively displaced from the ER, resulting in a neutralization of the estrogenic response.

### Histological observations of the gonads—left testis and ovary

The results of the present study show that in ovo exposure to EE_2_ leads to a reduction of the ovarian cortex in females. In males EE_2_ causes a significantly thickened testicular cortex with oocyte-like cells and female-like structure.

Furthermore, sole treatment with fulvestrant or tamoxifen does not significantly affect differentiation of male testes, which coincides with the studies of Salzgeber, Reyssbrion & Baulieu (1981) and Scheib & Baulieu (1981). Their experiments on chicken and quail show that tamoxifen especially affects female but not male gonadogenesis. Thus, tamoxifen-treatment especially disturbed the formation of the left ovarian cortex (Scheib, 1983). In the present study, exposure to 10 µg tamoxifen/g egg results in an altered distribution of the left ovarian cortex which might be based on the specific mechanism of action of tamoxifen. Tamoxifen is a partial agonist of the ER whose effect always depends on the specific tissue along with the intrinsic activity of tamoxifen. As ER alpha is primarily expressed in the gonadal cortex (Nakabayashi et al., 1998), its blockage at high doses might have contributed to the suppression of the cortical hypertrophy in the ovarian differentiation and its unbalanced dispersal. This might lead to the speculation that in higher doses tamoxifen might improve sterility of the respecting females, as the oogonia develop in the border between cortex and medulla (Gonzalez-Moran, 2011).

Since the ER is largely irrelevant for the normal differentiation of the male sex, fulvestrant and tamoxifen remain without statistically significant effect for males. However, the feminization of genetic males of domestic fowl and quail caused by estrogenic substances can be effectively compensated by antiestrogens (Samsel, Zeis & Weniger, 1982; Scheib, 1983) which agrees with the data of the present study. It can be assumed that both estrogenic and antiestrogenic substances compete for the binding site of the ER. Accordingly, a high estrogen-concentration leads to its preferential binding to the ER and results in the feminization of male gonads. With increasing concentration of the antiestrogen the ER is successively blocked. This results in a compensation of the feminizing effects as the ER and subsequent signal cascades are not affected by the estrogen.

## Overall Conclusions

The focus of the present work was on the study of the effects of the estrogenic compound EE_2_ as well as the antiestrogenic compounds fulvestrant and tamoxifen on embryonic sex development of chicken *(Gallus g. domesticus)*. In ovo exposure to EE_2_ resulted in a distinct feminization of genetic males which formed female-like cortex tissue in their left gonads. In addition, EE_2_-treatment resulted in a reduction of the percentage of seminiferous tubules. The antiestrogen tamoxifen influenced female sex differentiation and led to a size reduction of the left ovary and malformations of the ovarian cortex. In contrast, fulvestrant did not affect sexual differentiation in chicken in the tested concentration range. However, both antiestrogens were able to antagonize the feminizing effects of a potent estrogen in genetic males when administered simultaneously with EE_2_. Since both estrogenic and antiestrogenic substances induce concentration-dependent morphological alterations of the sex organs, the chick embryo can be regarded as a promising model for the identification of chemicals with estrogenic and antiestrogenic activity. However, it should be considered that the chick embryo is not necessarily sensitive to all classes of EDCs impacting the reproductive axis. Therefore, pending studies of androgenic and antiandrogenic compounds will provide more information about the suitability of the chicken embryo test for these classes of EDCs.

##  Supplemental Information

10.7717/peerj.5094/supp-1 Supplemental Information 1 Raw data of all measured values of the endpoints mentioned in the manuscript

## Additional Information and Declarations

### Data Availability

The following information was supplied regarding data availability: The raw data are provided in a Supplemental File.
